# Analysis of Wear Phenomena Produced by Erosion with Abrasive Particles against Fluoropolymeric Coatings

**DOI:** 10.3390/polym14214617

**Published:** 2022-10-31

**Authors:** Guillermo Guerrero-Vaca, Oscar Rodríguez-Alabanda

**Affiliations:** Department of Mechanics, University of Córdoba, Rabanales Campus, Leonardo da Vinci Building, Madrid-Cádiz Road, km 396, E-14071 Córdoba, Spain

**Keywords:** fluoropolymers, abrasives, sandblasting, polytetrafluoroethylene PTFE, fluorinated ethylene propylene FEP, perfluoroalkoxy alkane PFA

## Abstract

To date, PTFE, PFA, and FEP-based fluoropolymer coatings have proven unbeatable in many services due to their excellent chemical inertness, very low wettability, thermal resistance, high non-stick properties, and good applicability. In use, these coatings usually suffer service cycles with consequent deterioration, and it is of great interest to determine the intensity and type of wear caused in addition to the deterioration that occurs in their properties. In this work, the response of three polymeric coatings of interest applied to aluminum substrates, after being subjected to the action of abrasive particles of aluminum corundum, glass, and plastic projected under pressure, has been studied. During the application of a given wear cycle, the hardness, surface roughness, surface texture, and thickness of the coating have been measured, in addition to the slip angle and surface transmittance to analyze the evolution of each type of coating. The results allowed a concise evaluation of the performance of three fluoropolymeric coatings of great interest, differentiating the induced erosive wear phenomena and contributing complete information to facilitate the correct selection for users with practical application purposes and as a basis for future research work focused on advancements in this field.

## 1. Introduction

Coatings based on fluoropolymers, such as polytetrafluoroethylene (PTFE), fluorinated ethylene propylene (FEP), and perfluoroalkoxy alkane (PFA), can be applied to metals, ceramics, glasses, and textiles, among others, improving their surface properties. Their use has been extended to many applications, among others chemical [1], medical [2], automotive [3], textile, and alimentary [4]. After their use, these coatings wear and deteriorate, partially losing their quality [5].

These three fluoropolymers and their combination show extraordinary properties, which are summarized as an extreme chemical inertness [6] coupled with very low surface energy [7] and, therefore, non-stick properties [8], excellent electrical isolation [9], and high durability over a wide service temperature range. So, the correct selection is a determinant in terms of efficiency and eco-sustainability during its useful life cycle [10].

FEP and PFA polymers form a continuous film on the substrate. In both cases, the coating is applied by spraying or wetting, in the case of liquid resins, and via spraying or a fluidized bed in the case of powder application. Once the coating has been applied, the pieces must be polymerized in an oven between 350 and 380 °C and the fluoropolymeric layers can reach thicknesses between 40 and 125 μm [10].

Unlike previous ones, polytetrafluoroethylene (PTFE) resins are usually applied with liquid formulations. Typically, topcoats polymerize via thermal action between 390 and 425 °C and can typically reach thicknesses of 15 to 45 μm, but PTFE topcoats do not form a continuous layer and look like bond growth, which takes place in a powder metallurgical sintering process. Its hardness is relatively low, its surface tension is lower than that of FEP or PFA, and its processability limits the effective application thicknesses, generally lower than the thicknesses obtained in the case of fluoropolymers capable of forming films [11].

For the selection of a fluoropolymeric coating, in addition to its qualities, applicability, limitations, and costs, its durability under certain service conditions must be considered. The morphology of wear against the action of abrasive agents can facilitate the determination and evaluation of this durability. In this work, specimens have been subjected to controlled wear with three types of abrasives, and their effect on the different coatings has been studied. Works with similar objectives are found in the literature, such as those carried out for the removal of thermal barrier coatings focusing on ceramic top layers [12], TiB_2_ hard-coated steel substrates with diamond, alumina, and silicon carbide abrasives to determine their removal capacity [13], and the behavior of WC-Co nanostructured cermets against silica and alumina abrasives [14] or the response of hard metal (WC–5.7Co–0.3Cr) to abrasion with different particle sizes [15]. From another point of view, some previous research works that were consulted analyzed coatings of all kinds and origins: Multilayer composites, ceramic, and polymeric, in which wear phenomena were studied and, among them, due to abrasive particles [16,17,18]. In short, the experimental study of the behavior of a coating subjected to wear phenomena induced by the action of abrasives offers information of great interest to analyze its useful life and the loss of its properties, allowing an evaluation of its efficiency.

The present work is focused on the experimental study of the behavior of PTFE, PFA, and FEP-type fluoropolymeric coatings subjected to wear via the impact of abrasive particles. Thus, a set of experiments was addressed with the aim of evaluating the modification of the properties of these coatings and their behavior and evolution against wear. The experimental purpose was to evaluate the behavior of each of these coatings against induced erosion wear and identify the specific wear mechanisms involved in the process. This will contribute to the development of recommendations for the best selection for application purposes and provide a basis for future scientific work with this specific line of coatings.

## 2. Materials and Methods

### 2.1. Specimen Preparation

Three batches of 96 specimens of 30 × 30 × 1.1 mm aluminum alloy EN-AW 5754 H34 (Camebe, Castro Urdiales, Cantabria, Spain) were manufactured, and each batch was coated with a different fluoropolymer: PTFE, FEP, and PFA. The number of specimens was the result of studying the coatings after wear resulting from three different abrasives in 4 combinations of time and pressure and reiterating the tests 3 times per combination. This aluminum-magnesium alloy is of great interest because of its excellent behavior against corrosion, high hardness, and good machinability and weldability, and it is commonly used in the manufacturing of food products, refrigerators, storage tanks, or even shipbuilding, among many other uses in which the application of fluoropolymeric coating is common [19].

### 2.2. Coatings

The coatings studied are constituted by complex multilayer resins applied by specialists in the sector (Tecnimacor S.L, Córdoba, Spain) and are made up of binders, solvents, pigments, fillers, additives, and the fluoropolymer resin that constitutes the base and foundation of each coating: PTFE, FEP, and PFA. The main characteristics of the coatings used in this research are shown in Table 1.

The data in the table of characteristics indicate that the greatest thicknesses were achieved in the application of coatings with PFA, followed by FEP, and finally, PTFE, while the scratch hardness was higher in FEP, followed by PTFE, and finally, PFA. Roughness values between Ra = 0.77 μm and Ra = 0.83 μm were measured in all cases, which were very similar regardless of the type of coating. Considering wettability, the best behavior, that is, lower slip angles, was measured in the PFA, followed by FEP, and finally, PTFE.

Coatings were selected from the applicator’s portfolio that were representative of the qualities of each of the three fluoropolymers to be compared.

**Figure 1:**
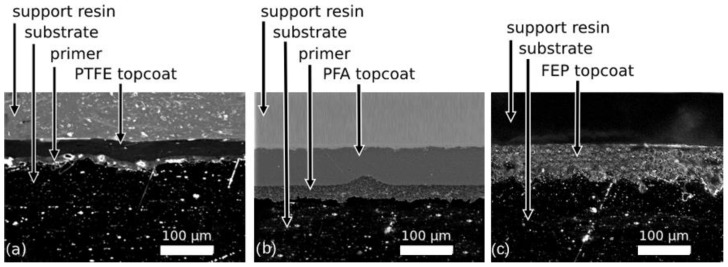
Sectional view of the coating: (**a**) TF-851 E PTFE, (**b**) TF-76521 PFA, and (**c**) TF-3531 FEP.

Thus, the PTFE coating is made up of a 3-layer system, an initial anchor or primer layer, followed by a second intermediate layer and a final layer, merged with each other. The PFA coating is made up of 2 layers, a primer layer and a final layer that has been applied in the form of PFA powder and that, after curing, generates a continuous film. The FEP coating is made up of 2 layers, likewise, an initial FEP layer and a final FEP top layer. In Figure 1, micrographs of the section of coated substrates described are shown.

**Figure 2:**
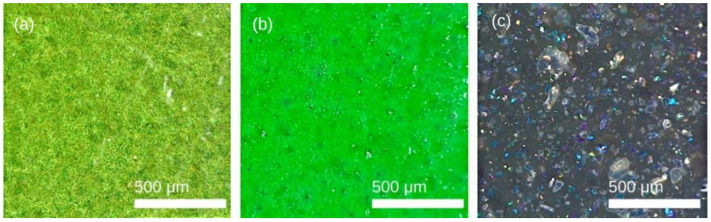
2D images of coatings obtained by confocal Leica DVM6 microscope: (**a**) PFA-base, TF-76521, (**b**) FEP-base, TF-3531, (**c**) PTFE-base, TF-851 E.

The aspect of the coatings object of study is shown in Figure 2, in the images of the supply state taken by a confocal microscope.

The appearance to the naked eye and that shown in the 2D images obtained show the well-differentiated aspect and particular morphological characteristics of each of these coatings. In one case, the appearance of a fairly uniform sheet can be seen, as is the case of PFA and FEP, and for PTFE, the appearance is more heterogeneous with a background that shows glosses and structures that reflect the metallic fillers that were included to improve the properties in these coatings [10].

### 2.3. Particle Projection, Equipment, and Abrasives

**Figure 3:**
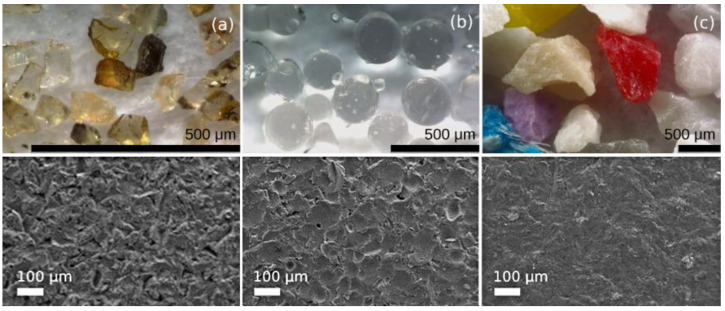
Morphology of the abrasives by Leica DVM6 confocal microscope: (**a**) Brown corundum, (**b**) glass microspheres, (**c**) polymeric-plastic particles, and SEM images of sandblasted aluminum substrates.

Three types of abrasives were used for the experimental tests, of which the properties and characteristics are indicated in Table 2. Figure 3 shows the microscope images of the bare aluminum substrate after the action of each one of the different types of abrasives were projected with pressure against its surface.

The brown corundum, which was supplied by the MPA company (MPA, Cornellá de Llobregat, Barcelona, Spain), is marketed as ALODUR^®^ RBT 9. The glass microspheres were provided by the Potters Group company (Potters Industries, Phoenixville Pike Malvern, PA, USA), with the trade name Spheriglass^®^ Solid Glass Microspheres, and the used grade is 1821. The polymeric-plastic shot was from the Abshot Tecnics S.L. company (Abshot Tecnics S.L., Cervelló, Barcelona, Spain) as type III granules, also known as multicolor melamine. These abrasives were selected due to the different qualities they possess for the industrial treatment and cleaning of these coatings and the diversity of behaviors they can produce.

For the projection of the particles, suction equipment (venturi effect) was used, namely, the Sand Blast Cabinet CAT-990 (Aslak S.L., San Quirze del Valles, Barcelona, Spain). The projection nozzle was Ø 6.5 mm. The projection was carried out at a distance of 200 mm from the substrates and 90° from the covered zone with a pressure of 0.4 MPa during time intervals of 4 s, 8 s, and 12 s.

The fluoropolymeric coatings were damaged and worn after the projection of the different abrasives, and each type of abrasive produced very diverse effects that stimulated different behaviors of the coatings.

### 2.4. Properties Analyzed after the Wear Process

The thickness of the coatings was determined with a Leptoskop 2042 portable thickness gauge (Karl Deutscht, Wuppertal, Germany), and 5 measurements were made on the same specimen. The roughness Ra and Rz were determined with the Mitutoyo SJ-201 roughness meter (Mitutoyo, Kanagawa, Japan) after 3 measurements on different points of the specimen. With the Leica DVM6 and DCM8 confocal microscopes (Leica microsystems, Wetzlar, Germany), the 3D textures for the specimens subjected to wear were determined.

The coating hardness was measured with a durometer rod also known as an SP0010 TQC hardness pencil (TQC Ltd., Nottingham, UK) by dragging a Ø 1 mm tungsten carbide tip over the coated surface at a constant pressure regulated by a spring. The final data were obtained after repeating the measurement 3 times.

The wettability properties were measured by obtaining the sliding angle (SA). The equipment consists of an inclinometer that allows one to measure the angle of fall of a drop deposited on a horizontal surface that is tilted by means of a motorized system at a constant angular velocity of 10°/min. This apparatus mounts a goniometer with 0.05° of appreciation and, when the complete sliding movement of the water drop occurs, the angular value can be read. For this purpose, 50 and 100 µL water droplets were applied onto the surfaces of interest.

The absorbance of the samples subjected to wear was studied with a Bruker Tensor 27 FT-MIR machine (Bruker, Billerica, MA, USA). The spectra were obtained with a spectrometer in attenuated total reflectance (ATR) mode. It was a spectrometer with Cesium Iodide (CsI) beam splitters, a Deuterated Triglycine Deuterated Sulphate (DTGS) detector, and a single ATR reflection with a Germanium (Ge) crystal.

## 3. Results and Discussion

### 3.1. Wear Phenomena

The removal of a coating via abrasive projection is a relatively complex phenomenon that has three phases: Delamination starts at the beginning of the impact, followed by a buckling of the film due to the greater radial compressive stresses that usually come from the penetration of the particle into the coating, and finally, total delamination [20]. In addition, two types of dominant mechanisms can be identified, one due to plastic deformation and the other due to brittle fracture [21]. In the different cases in this work, and due to the nature of the abrasives and interaction with the polymeric-thermoplastic coatings, wear occurs via plastic deformation and, in turn, by mechanical shearing and strain hardening caused by impact phenomena.

On the other hand, abrasion resistance is usually measured by the Taber test procedure described by ASTM D1044. The abrasion resistance of unfilled semi-crystalline polymers is linked to the degree of crystallinity and this, in turn, is related to the molecular structure and weight of the resin and its processing. In the case at hand, deposited coatings are complex and may contain different types of charges or fillers. These coatings are made up of multiple layers, and their processing differs between each type in such a way that the results reported in the literature for unfilled materials are not consistent with the coatings put into service [22]. In any case, it is known that the hardness of PTFE, FEP, and PFA fluoropolymers without fillers are between 56 and 59 Shore D, according to ASTM D2240 at 23 °C, with a wear resistance index in the Taber test of 9–11 mg/1000 cycles. These values are worse than those corresponding to an engineering polymer such as polyamide (2–4 mg/1000 cycles) but better than high-density polyethylene (25 mg/1000 cycles) [22].

### 3.2. Wear Produced by Brown Corundum Particles Projection

In the wear produced by abrasives such as brown corundum, plastic deformation is produced, generated by the impact and the shear stress of the particles that stick, penetrate, and expel the coating [23,24]. This type of wear can be associated with abrasive impact and shear wear. These particles have a very angular morphology and are known to have a high influence on abrasive wear and removal rates [25]. Brown corundum has a high hardness, 9 on the Mohs scale, and a relatively high specific weight in the range of 3.9–4 g/cm^3^. Thus, Table 3 shows the change in the main properties of the coatings after being processed with brown corundum projected at 0.4 MPa.

From the results of the wear tests with the indicated abrasives, it was observed that the coating with the lowest loss of properties is the PFA-based coating (TF-76521). In this case, the thickness decreases by 11.6%, (initial thickness of 115.9 μm), the scratch resistance (hardness) of the coating increases by 12.2%, the roughness Ra increases from 1.5 µm initially to 2.5 µm after projection, and the slip angle (SA), which describes the water repellency, worsens from an initial value of 9.6° to 25.6°. Thus, the initially hydrophobic surface became hydrophilic.

In short, the PFA coating hardens with wear, loses thickness slightly, increases its roughness, and significantly worsens its water-repellency capacity, although the loss of these properties is less pronounced than in PTFE and FEP, which were subjected to the same treatment of wear.

**Figure 4:**
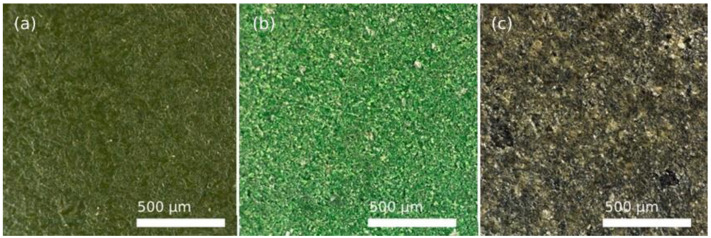
2D images of the coatings after wear process during 4 s projecting with brown corundum particles at 0.4 MPa, obtained with Leica DVM6 confocal microscope: (**a**) PFA, (**b**) FEP, and (**c**) PTFE.

Particularly interesting is the 12.2% increase in the scratch resistance of the PFA coating, since this is a unique phenomenon that does not occur in either FEP or PTFE and may be due to the significantly higher initial layer thickness, 115 μm for PFA compared to 78 μm for FEP and 30 μm for PTFE. It is known that the hardness of these polymers is similar and so are their mechanical properties [3], and the phenomenon may be linked to the compressive effect produced by spraying on a thick layer of PFA powder with relatively low crosslinking as is the case of the PFA. The application of this coating was performed using very fine particles of PFA powder that are deposited on the primer and that, after curing, stretch and form a low-hardness top sheet. It is likely that the abrasive particle projection compacts the polymer and, ultimately, the PFA layer, therefore increasing the hardness. In the case of PTFE and FEP, the coating layers are thinner, and no final powder layer is applied, with the possibility of this compression effect reaching even the harder primer layers below. Figure 4 shows the visual appearance of coatings after wear with brown corundum.

**Figure 5:**
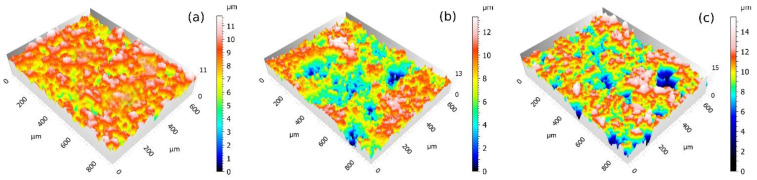
3D captures (×20 confocal lens, Leica DCM8 high-definition microscope) of the coatings after projection during 4 s with brown corundum at 0.4 MPa: (**a**) PFA, (**b**) FEP, and (**c**) PTFE.

In the observation of the specimens, PFA shows a homogeneous appearance without failure, although slight changes in the texture and an increase in its mean roughness Ra have been measured. On the contrary, greater degradation was detected for FEP and PTFE coatings. Particularly evident is the case of PTFE, since losses of coating material have been clearly observed, even leaving the substrate uncovered. In the 3D captures of the textures that are shown in Figure 5, this wear effect has been revealed by the greater height difference in the Z axis in the PFA samples, especially in the case of PTFE.

In summary, the FEP and PTFE-based coatings suffered notable decreases in thickness, producing a decrease in hardness and worsening water repellency more intensely mainly due to the lifting and shedding of the coating. In conclusion, their use may be rarely or very rarely recommended when the wear of the surface shows an abrasive origin mainly caused by impact and shearing, which is what is generated by the brown corundum particles.

**Figure 6:**
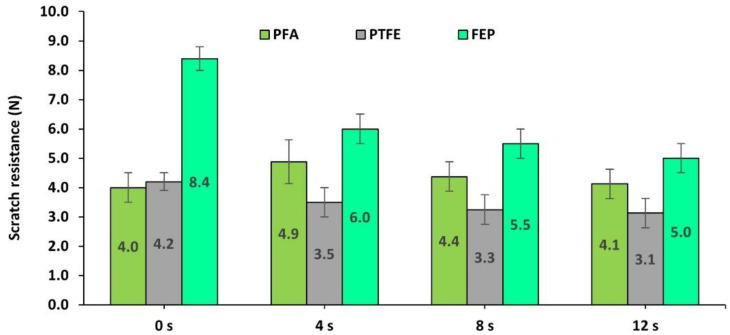
Scratch resistance (N) of the coatings based on PFA, PTFE, and FEP, after 0, 4, 8, and 12 s subjected to the projection of brown corundum particles at 0.4 MPa.

On the other hand, it is of interest to determine the rate of loss of properties that are reached in the different fluoropolymers. The wear tests were carried out from 4 to 8 and 12 s. To do this, the scratch resistance was analyzed as one of the properties most closely related to the integrity of coatings. Figure 6 shows the variation of this response.

It is observed that the coating with the highest scratch resistance after 12 s is FEP, followed by PFA and PTFE. The PFA coating, although it initially showed an increase in hardness, ended up with relatively low values of 4.1 N. The PTFE had its hardness modified to a lesser extent from 4.2 to 3.1 N.

In short, analyzing the response to abrasion with brown corundum that is associated with abrasive wear due to impact and shearing, the coatings with the best behavior were PFA fluoropolymers followed by FEP, with PTFE being the least recommended. However, when the coatings are subjected to prolonged wear cycles, the FEP coating shows greater integrity in prolonged use.

### 3.3. Wear Produced by Glass Microspheres Projection

The wear produced by the glass microspheres causes a preponderant impact effect that generates a compressive effect and deformation of the coating due to its low angular morphology, although it is highly probable that a part of the microspheres will break on the surface of the material due to the impact, and sharp and broken edges can erode the material [26]. Thus, the traces of craters on the coating surface worn by this type of abrasive particles are characteristic of this phenomenon of impact and plastic deformation [27].

Table 4 shows the modifications of the properties of the coatings tested with glass microspheres.

The analysis of the results gave us several consequences. Firstly, the polymer that was most affected in its properties is the PFA-based polymer, with a 14.6% decrease in thickness, a 12.5% decrease in scratch resistance, and a substantial increase in roughness at 3.07 μm. On the other hand, the wettability properties were less affected by this type of wear, and, in any case, the best results were obtained for PTFE showing SA values of 13.9°, obtaining relatively similar data to PFA (16°), but FEP was notably higher (27.0°).

**Figure 7:**
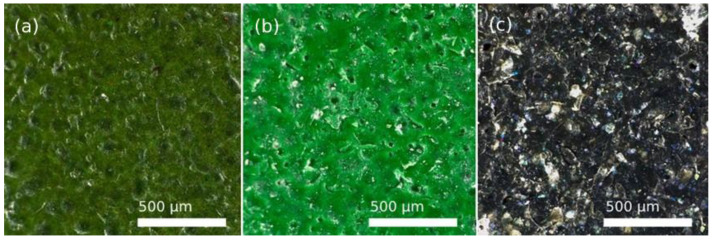
2D images of the coatings after wear process during 4 s by the projection of glass microspheres at 0.4 MPa, obtained with Leica DVM6 confocal microscope: (**a**) PFA, (**b**) FEP, and (**c**) PTFE.

Figure 7 shows the appearance of PFA, FEP, and PTFE fluoropolymers after shear and impact abrasion wear produced by glass microspheres.

**Figure 8:**
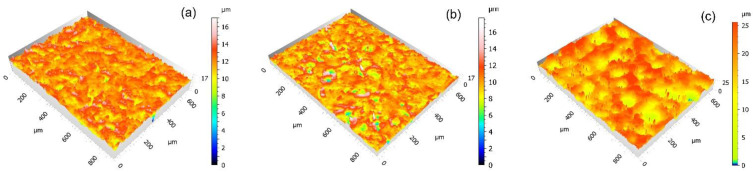
3D captures (x20 confocal lens, Leica DCM8 high-definition microscope) of the coatings after projection during 4 s with glass microspheres at 0.4 MPa: (**a**) PFA, (**b**) FEP and (**c**) PTFE.

2D images shown in Figure 6 evidence that the impacts of the glass microspheres caused the formation of soft craters in all coatings showing similar shapes and sizes for PFA and FEP, but slightly larger and deeper in PTFE. In none of the cases was the substrate affected. This deformation effect is manifested in the 3D captures shown in Figure 8.

**Figure 9:**
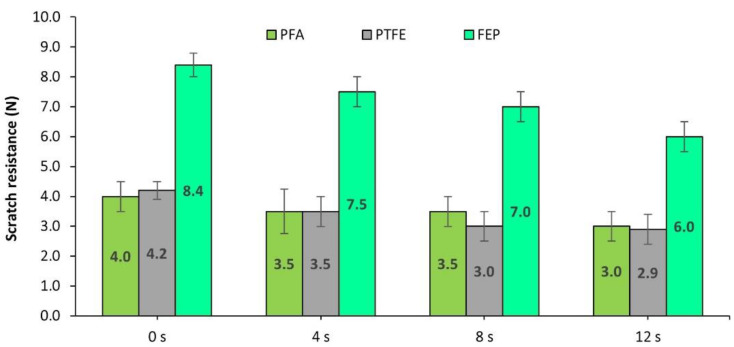
Evolution of the scratch resistance (N) shown by PFA-, PTFE-, and FEP-based fluoropolymeric coatings after 0, 4, 8, and 12 s wear processes via the projection of glass microspheres at 0.4 MPa.

On the other hand, the effect of wear induced for 4, 8, and 12 s on scratch resistance was analyzed, as was performed in the previous case. Thus, the graph in Figure 9 shows that, in prolonged wear, the FEP coating reaches the highest values of hardness with a final value of 6.0 N. The severe condition shown by the PFA and PTFE coatings is noteworthy, since, in addition to having initial resistance values significantly lower than FEP, after the wear process, their resistance to scratching is significantly reduced and shows very inadequate behavior against this type of wear.

**Figure 10:**
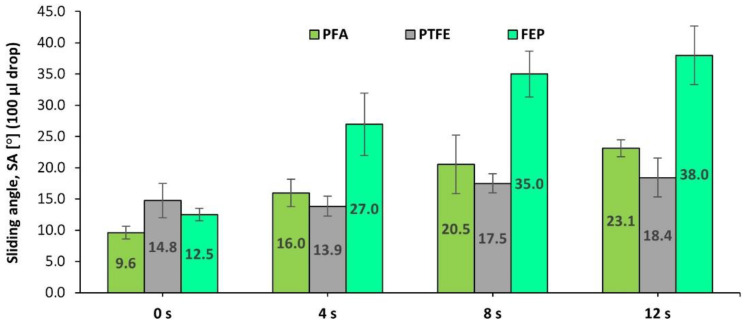
Evolution of the sliding angle (SA) of PFA-, PTFE-, and FEP-based fluoropolymeric coatings after 0, 4, 8, and 12 s wear processes via the projection of glass microspheres at 0.4 MPa.

Further, it is interesting to analyze the variation of wettability properties since, in this case, significantly better values have been observed compared to those measured after impact and shear wear with brown corundum particles, as shown in Figure 10.

In this case, it can be clearly seen that, for the effects of this type of wear and from the point of view of water repellency, the best coating is PTFE, which maintained SA values much lower than 20° after the whole wear process.

In short, the coating with the greatest durability in the case of wear of a compressive plastic deformation nature has been the PTFE-based coating, while the FEP showed a progressive worsening in a reasonable range of values. However, PFA’s wettability qualities worsen exponentially with respect to its exposure to this type of abrasive agent. In any case, it must be considered that the higher relative thicknesses of the FEP coating could be advantageous in the case of applications in which the integrity of the coating is required after a long duration of wear.

### 3.4. Wear Produced by Plastic-Polymeric Particles Projection

On this occasion, melamine plastic particles with a low specific weight, from 1.2 to 1.5 g/cm^3^, and with an average hardness of 4 on the Mohs scale, were used. The type of wear of this abrasive is known for its use of ensuring the integrity of the substrate [28] and is commonly used for paint stripping on aircrafts [29]. The effect it produces functions via delamination [30], such as drawing, but is less influenced by impact effect wear and shear cutting. Table 5 shows the data on the variation of properties of the coatings under study after projection with this type of particle.

In this case, after wear with this type of abrasive, the coating that showed the best behavior is PFA. This can likely be justified by the higher thickness of the coating and its structure. PFA has a high layer of cross-linked powder on the surface forming a film. This polymeric layer will likely cushion some of the impacts of the abrasive. The second-best-performing coating was FEP, and the worst was PTFE, which suffered a very intense weakening of its properties.

In this case, the final roughness for all the coatings was, surprisingly, in the environment of 8–9 μm, between 2 and 3 times higher than that obtained with the other abrasives. Water repellency worsened as expected but, again, more intensely in PTFE. The loss of thickness and scratch resistance was particularly significant in PTFE.

Considering that the hardness of this type of abrasive is medium, it is conceivable that the phenomenon of wear may be linked to the fact that the abrasive is more tenacious and relatively more ductile and then, after the impact, it will be less incisive, generating a stretching effect on surfaces of greater elasticity and relatively thicker as is the case of PFA and FEP. On the contrary, in coatings with lower layer thickness, which also present heterogeneity in its matrix due to the load of metallic fillers, as is the case of PTFE, the elasticity of the coating is relatively low, and the effect is much greater.

**Figure 11:**
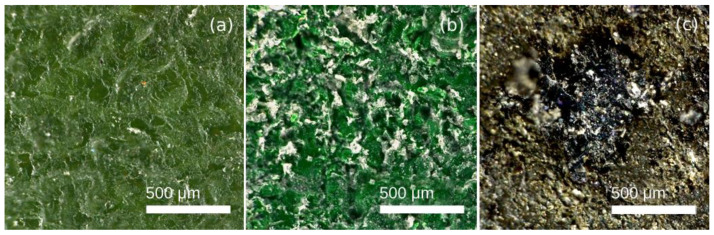
2D images of the coatings after wear process of 4 s by the projection of plastic-polymer at 0.4 MPa, obtained with Leica DVM6 confocal microscope: (**a**) PFA, (**b**) FEP, and (**c**) PTFE.

Figure 11 shows the appearance of PFA, FEP, and PTFE fluoropolymers after abrasive wear with polymer particles.

**Figure 12:**
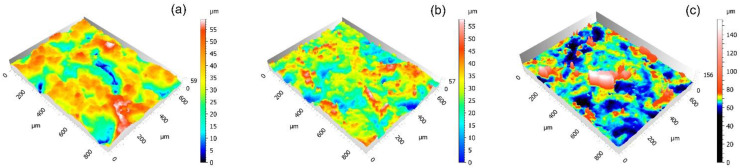
3D topographic captures (×20 confocal lens, Leica DCM8 high-definition microscope) of the coatings after projection of 4 s with plastic particles at 0.4 MPa: (**a**) PFA, (**b**) FEP, and (**c**) PTFE.

The naked-eye analysis of the specimens corroborates the numerical results. Figure 11a shows a deformed and stretched coating in the case of PFA, while the FEP coating seems clearly more affected by the same effect and, in the case of PTFE, severe damage is observed, even affecting the metallic substrate. These differences in the behaviour of the different coatings were also revealed in the 3D captures shown in Figure 12 Particularly, in the case of PTFE (Figure 12c), the 3D view shows large areas of the substrate that have lost the coating, leaving it bare (dark colour), and the white-red peaks represent coating traces about to completely detach from the substrate.

**Figure 13:**
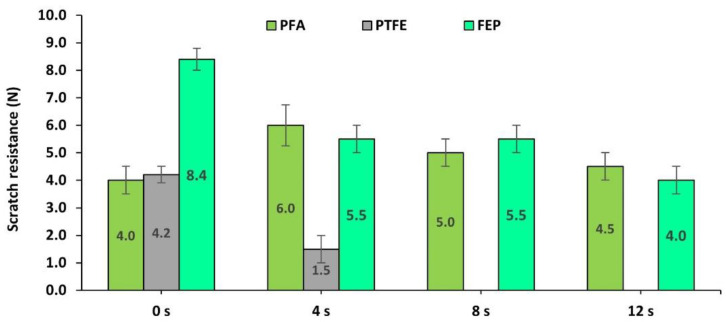
Evolution of the scratch resistance (N) shown by PFA, PTFE, and FEP after 0, 4, 8, and 12 s wear processes via the projection of plastic-polymer particles at 0.4 MPa.

Finally, a summary of the results on the effect on scratch resistance after cumulative projection for 0, 4, 8, and 12 s of polymeric abrasive is presented in Figure 13.

In this case, the progression of wear shows that the hardness of the PTFE layer dropped dramatically, in such a way that, at 8 and 12 s, the PTFE layer disappeared, revealing the bare substrate. This fact has been evidenced in a rapid decrease of more than 100% in the scratch resistance of the PTFE coating with only 4s of action of the abrasive, from 4.1 N to only 1.5 N, completely eliminating the PTFE coating layer even before reaching 8s in the wear process. However, in the case of FEP and especially PFA, the coating still maintained a certain resistance to scratching even with the longest wear time. Once again, the PFA coating has shown that an increase in hardness may be due to the compression of the coating layer. Thus, the scratch resistance has been increased initially remaining above the initial value of this coating, even after the entire wear process.

In descriptive terms, the wear phenomenon produced by the action of abrasive particles projected under pressure against a surface is defined as an erosion phenomenon [20]. Upon analyzing the wear mechanisms occurring in the cases described in this study, it should be noted that previous works always differentiate between the two types of mechanisms involved in this type of wear: Plastic deformation and brittle fracture [15,20,21].

There are several key factors that will condition the type of mechanism in each case. In general, the coefficient of friction between the particle and the attacked surface will always be decisive, since low friction at the interface leads to a greater shear effect and, normally, a higher wear rate. In addition, the shape, particle size, and the direction of their projection with respect to the attacked surface are also determining factors. Thus, Zouari et al. [20] modeled the process of removal of polyurethane paint via the projection of steel particles and concluded that, when this type of coating is attacked with particles projected under pressure and in a direction normal to the surface, radial shear stresses accumulate as the material hardens via cold deformation due to impact until delamination and coating failure begin. Along the same lines, the experience carried out by Thakare et al. [15] showed that the effect of abrasive SiC particles used to abrade WC-based hard coatings was highly dependent on their size, shape, and also on their hardness. On the one hand, they defined that the sharpness of an abrasive particle is a function of the average angle of its edges and its size, a quality defined by the parameter called the spike value. In their experiment, they showed that abrasive particles with a higher spike value have a higher abrasive power. However, they also defined that the degree of wear of a surface subjected to erosion depends, to a large extent, on the ratio between the hardness of the abrasive and the hardness of the attacked surface.

In this sense, it should be noted that the brown corundum abrasive particles used in the present work have a relatively small size and high hardness compared to the hardness of the attacked coatings, but they present important sharpness. Consequently, wear in which the shear mechanism predominates has been observed, characterized by a shearing effect that caused a significant but homogeneous degree of wear on the FEP and PTFE coatings.

In the case of glass microspheres, they have a relatively high hardness compared to the attacked polymers. With a size similar to that of brown corundum particles, they have a smooth shape and no edges, resulting in no sharpness. These qualities imply wear produced eminently by plastic deformation effects and hardening by compressive deformation that, in none of the cases studied, lifted the coating, although it slightly degraded the thickness and resistance to scratching.

Unlike brown corundum or glass microspheres, the plastic-polymer particles present a larger size and pronounced edges, but a much lower hardness. These physic-geometric characteristics imply, on the one hand, plastic deformation of the particles and the coating and, on the other hand, the shear effect because of the sharpening of the particles and the shear stress accumulated by the impacts. In our case, this effect caused strong wear of the FEP coating and essentially the total degradation of the PTFE coating, while the PFA coating showed better behavior.

### 3.5. FTIR Analysis

**Figure 14:**
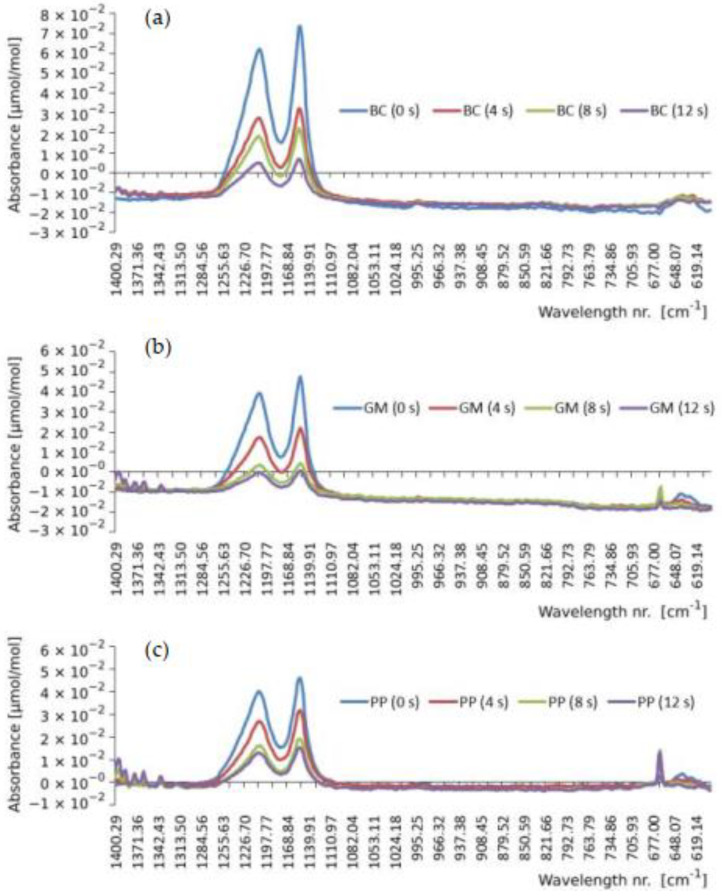
Evolution of the absorbance intensity of PFA coating after 0, 4, 8, and 12 s of wear via the projection of (**a**) brown corundum (BC), (**b**) glass microspheres (GM), and (**c**) plastic-polymer (PP).

In this analysis, the PFA fluoropolymer was studied as the most representative case of the behavior of the coatings object of this study. Figure 14 shows the FTIR spectra corresponding to the wear progression of this polymeric coating subjected to erosion by each type of abrasive used.

The wavelength values of 1209 cm^−1^ and 1153 cm^−1^ appear in the literature as relatively high absorbance “peaks” corresponding to the C-F bonds characteristic of fully fluorinated fluoropolymers. On the other hand, although weaker, there is a peak at a wavelength value of 994 cm^−1^, which is associated with the C-O-C vibrations of perfluoro alkoxy vinyl ether, i.e., PFA [31]. On the other hand, the variation of absorbance generated by the action of each type of abrasive projected at 0.4 MPa for 0 s, 4 s, 8 s, and 12 s against the PFA coating has been analyzed. Thus, it has been observed that the absorbance intensity values are consistent with the wear tests, as they decrease as the polymer layer decreases due to layer loss and likely due to the interaction with the abrasive.

## 4. Conclusions

Three types of fluoropolymeric coatings, PTFE, FEP, and PFA, were analysed under wear with abrasive particles of different characteristics: Brown corundum, glass microspheres, and plastic particles.

These coatings have shown different behaviours depending on the type of wear induced by abrasion. The results can allow researchers, users, and applicators to be guided in the selection of the best alternatives when the type of wear in service is similar to those determined in the investigation. In detail, it can be concluded that:

- In the type of wear due to impact and shearing (brown corundum), the best option is the PFA fluoropolymer coating, and the FEP-based coating is second.
- When the coatings have been subjected to prolonged wear cycles with brown corundum, the FEP coating showed greater integrity for this use. PTFE should not be used for this type of wear.
- In the type of wear generated by the compressive effect (glass microspheres), PTFE-based coatings have shown the best performance. As a second option, the FEP coating could be selected.
- The higher relative thicknesses of the FEP coating could be more advantageous than the lower thickness of PTFE for applications in which the integrity of the coating is required after a long duration of exposure to the type of wear induced by the pressure application of glass microspheres.
- In the type of wear due to stretching until delamination (polymeric particles), PFA has shown the best performance followed by FEP.
- Even in applications with long wear times induced by plastic particles, the PFA coating has shown the best behaviour. PTFE should not be used in these conditions since is very sensitive to this type of wear.
- The sliding angle (SA) was significantly worsened by the effects of abrasion that separates the coatings from the substrate by shearing or stretching (brown corundum and plastic particles).
- On the contrary, the hydrophobic yield remained much less altered when the effect of wear was reduced to a purely compressive effect (glass microspheres).
- FTIR analysis has identified the fluoropolymers and shows results consistent with the levels of wear associated with the blasting parameters.

Finally, for the use of a fluoropolymer coating for applications in which these types of wear mechanisms may be associated, the best-performing coatings are, by far, those based on PFA when shear abrasion mechanisms combined with plastic deformation predominate, and those based on FEP in those cases in which the wear mechanism is eminently induced by plastic deformation. The present work contributes experimental results that constitute a scientific basis to be able to approach future works for the modeling of phenomena of wear by abrasion revealed by the use of the type of abrasives and coatings.

## Figures and Tables

**Table 1:** Characteristics of the PFA, FEP, and PTFE fluoropolymeric coatings used.

Reference	Resin	RAL Colour	Thickness (μm)	Scratch Resistance (N)	Roughness Ra (μm)	Sliding Angle SA (°)
TF-76521	PFA	130 40 20	115.9 ± 14.7	4.0 ± 0.5	0.77 ± 0.12	9.6 ± 1.0
TF-3531	FEP	140 40 30	77.9 ± 12.8	8.4 ± 0.4	0.81 ± 0.09	12.52 ± 1.0
TF-851 E	PTFE	220 20 05	30.11 ± 5.4	4.2 ± 0.3	0.83 ± 0.10	14.75 ± 2.7

**Table 2:** Characteristics of the abrasives used in the experiments.

Abrasive	Mohs Hardness	Grain Size (μm)	Specific Weight (g/cm^3^)
Brown corundum	9	106–150	3.9–4.0
Glass microspheres	6	300–200	2.5
Polymeric plastic particles	4	850–600	1.5–1.6

**Table 3:** Variation in the properties of the coatings between the initial state and after 4 s of wear via projection with brown corundum particles (CM).

Coating	% Thickness Variation (μm)	% Scratch Resistance Variation (N)	Roughness Ra (μm)	Sliding Angle SA for 100 µL Drop (°)
PFA	−11.6	+12.2	2.70	25.6
FEP	−13.1	−28.7	1.47	>30
PTFE	−23.9	−19.1	1.69	21.4

**Table 4:** Properties of fluoropolymeric coatings after 4 s of wear by glass microspheres at 0.4 MPa.

Coating	% Thickness Variation (μm)	% Scratch Resistance Variation (N)	Roughness Ra (μm)	Sliding Angle SA for 100 µL Drop (°)
PFA	−14.6	−12.5	3.07	16.0
FEP	−11.2	−10.9	1.95	27.0
PTFE	−10.2	−11.6	2.75	13.9

**Table 5:** Properties of fluoropolymeric coatings after 4 s of wear by plastic-polymeric particles projected at 0.4 MPa.

Coating	% Thickness Variation (μm)	% Scratch Resistance Variation (N)	Roughness Ra (μm)	Sliding Angle SA for 100 µL Drop (°)
PFA	−8.08	+33.3	7.74	23.03
FEP	−8.94	−34.52	7.73	23.31
PTFE	−70.11	−58.54	9.33	>30

## Data Availability

Not applicable.
